# Antibacterial Activity of Coumarins and Carbazole Alkaloid from Roots of *Clausena anisata*

**DOI:** 10.1155/2019/5419854

**Published:** 2019-02-03

**Authors:** Dandena Tamene, Milkyas Endale

**Affiliations:** Department of Applied Chemistry, Adama Science and Technology University, P.O.Box 1888, Adama, Ethiopia

## Abstract

*Clausena anisata* is one of the medicinal plants used traditionally for treatment of parasitic infections, irritation (boils, ringworm, and eczema), flatworm infestations, influenza, abdominal cramps, and constipation. Phytochemical screening test of dichloromethane/methanol (1 : 1) roots extract revealed the presence of flavonoids, phytosterols, coumarins, phenols, alkaloids, tannins, terpenoids, and free reducing sugars and the absence of saponins. Silica gel column chromatographic separation of the dichloromethane/methanol (1 : 1) extract afforded a carbazole alkaloid derivative of heptazoline (**1**) and three coumarins (**2**–**4**), including the known coumarins imperatorin (**3**) and chalepin (**4**). Structures of the compounds were elucidated by spectroscopic techniques (IR, ^1^H NMR, ^13^C NMR, and DEPT-135). Antibacterial activity of the crude extracts and isolated compounds was screened using agar diffusion method against strains of *Staphylococcus aureus*, *Escherichia coli*, *Pseudomonas aeruginosa*, and *Bacillus substilis*. The results of antibacterial test revealed derivative of heptaphylline (**1**) and imperatorin (**3**) exhibited comparable antibacterial activity against *S. aureus* and *B. substilis* (14 and 13 mm zone of inhibition, respectively) to that of ciprofloxacin (15 mm zone of inhibition) at a concentration of 20 *µ*g/mL. Chalepin (**4**) revealed more antibacterial activity against *B. substilis* (16 mm zone of inhibition) compared to ciprofloxacin (15 mm).

## 1. Introduction

**Figure 1:**
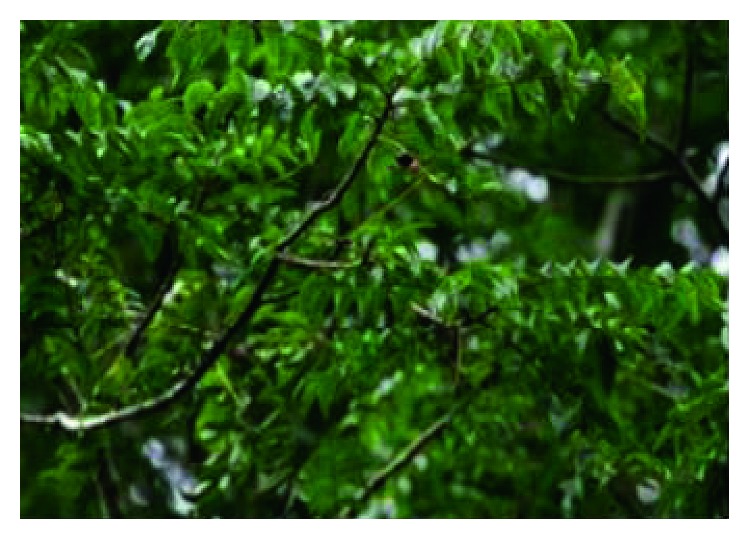
*C. anisata* (olmaa'ii) (Photo taken by Dandena Tamene on March, 2018).

Medicinal plants have been used for treatments of various diseases since antiquity and still play an important role to cover the basic health needs in the developing countries [1]. Most of the people in rural and urban areas of the developing world depended on the medicinal plants for the treatment of infectious diseases. *Clausena anisata* (Willd) Hook. f. ex Benth (Figure 1), known by local name “olmaa'ii” in Ethiopia (Afan oromo language), belongs to the family Rutaceae and is widely used in various parts of Africa for the treatment of bacterial and fungal infections of the skin including boils, ringworm, oral thrush and eczema [2], and malaria [3]. We hereby present a comprehensive phytochemical analysis and antibacterial evaluation of the roots extract of *Clausena anisata*.

## 2. Experimental Section

### 2.1. General

Column chromatographic separation was carried out on silica gel 60 (70–230 mesh size, Merck). Thin-layer chromatography was done on silica gel 60 F-254, 0.25 mm thick layer on aluminum sheets for detection of spots. Samples were applied on column by either adsorbing on silica gel or dissolving in appropriate solvent. The infrared (IR) data were recorded on a Perkin Elmer model FT-IR spectrometer (400–4000 cm^−1^) using KBr disks. The ^1^H NMR, ^13^C NMR, DEPT-135, spectra were recorded using Bruker Avance 400 MHz spectrometer using TMS as internal standard. Chemical shift values for all NMR data are reported in parts per million (ppm) relative to internal standard. All the chemicals used were of analytical grade from RanChem General Trading PLC, Addis Ababa, Ethiopia.

### 2.2. Plant Material Collection and Identification

The roots of *C. anisata* (Willd) Hook. f. ex Benth were collected from the Oromia region, west Wollega zone, which is 541 km west of Addis Ababa on March 2018. The plant was identified by the botanist Shambel Alemu, National Herbarium of Ethiopia, Addis Ababa University. The collected roots of the plant were thoroughly washed using distilled water to remove dirtiness, air-dried in the shade, and stored. The dried plant roots were cut into small pieces, air-dried, and ground into a fine powder.

### 2.3. Extraction

The pulverized powders (500 g) were soaked with dichloromethane/methanol (1 : 1) for 72 hr with occasional shaking. The extract was filtered and concentrated using a rotary evaporator at 40°C to give crude 8.7 g extract (1.74% yield).

### 2.4. Isolation of Compounds

Crude extract (8.5 g) was adsorbed on equal amount of silica gel and subjected to silica gel column chromatographic separation (150 g silica gel) and eluted with increasing gradient of ethyl acetate in *n*-hexane. A total of 65 fractions were collected. Fractions that showed similar *R*_f_ values and characteristic color on TLC were combined. Fractions 11–12 showed yellow spot under UV light having the *R*_f_ value of 0.56 in *n*-hexane/ethyl acetate (8 : 2) solvent system. After concentrating, the solid material left was repeatedly washed with *n*-hexane to yield compound **1**. Fractions 19–25 were combined since all fractions showed single spot and similar *R*_f_ values. After concentrating, the solid was washed repeatedly with *n*-hexane to afford compound **2**. Fraction 29–34 (16 mg) were combined on the basis of TLC profile and showed a single red colored spot on TLC using *n*-hexane: EtOAc (6 : 4) as a mobile phase. After concentrating, the solid was washed repeatedly with *n*-hexane to afford imperatorin (3). Fractions 46–50 (27 mg) were combined on the basis of TLC profile and showed one spot on TLC using *n*-hexane: EtOAc (5 : 5) as eluent. After concentrating, the solid was washed repeatedly with *n*-hexane to afford chalepin (4).

### 2.5. Phytochemical Screening Test

Phytochemical screening tests were performed using standard protocols in literature [4–8].

#### 2.5.1. Test for Terpenoids

Crude extract (0.5 g) was dissolved in 5 mL of methanol, and 2 mL of the extract was treated with 1 mL of 2,4-dinitrophenyl hydrazine dissolved in 100 mL of 2 M HCl. The formation of the yellow-orange color confirms the presence of terpenoids [4].

#### 2.5.2. Test for Alkaloids

Crude extract (0.3 g) was mixed with 2 mL of concentrated hydrochloric acid. The mixture was then filtered and mixed with a small amount of amyl alcohol at room temperature. Few drops of Dragendroff's reagent (solution of potassium bismuth iodide) were added to the acid layer and a reddish brown precipitate was observed [5].

#### 2.5.3. Test for Tannins (Gelatin Test)

Small quantity of the extract was mixed with water and heated on water bath. To the extract, 1% gelatin solution containing sodium chloride was added. Formation of white precipitate indicates the presence of tannins [6].

#### 2.5.4. Test for Saponins (Froth Test)

Crude extract (0.5 g) was dissolved in 5 mL distilled water. The mixture was shaken vigorously and stable persistent froth was obtained [7].

#### 2.5.5. Test for Flavonoids

Ethyl acetate (10 mL) was added to the crude extract (0.5 g) and heated for 3 min using steam bath. The mixture was filtered and the filtrate (4 mL) was mixed with 1 mL of dilute ammonia solution. Formation of intense yellow color ratifies the presence of flavonoids [4].

#### 2.5.6. Test for Anthraquinones (Bontrager's Test)

Crude extract (0.5 g) was taken into the first test tube, and 5 mL chloroform was added while shaking for 5 min. The extract was filtered to the second test tube and shaken with an equal volume of 100% ammonia solution, to obtain a pink violet or red color in the ammoniacal layer (lower layer) [4].

#### 2.5.7. Test for Phenols

Few drops of 2% of FeCl_3_ were added to the crude extract (0.5 g), and the formation of bluish green to black color indicates the presence of phenols [8].

### 2.6. Antibacterial Testing

#### 2.6.1. Preparation of Discs Containing Extracts

The same concentrations of 20 *µ*g/mL were prepared from the extract, isolated pure compounds, and the standard. The concentration was incorporated into sterile agar-disc diffusion and dried at 37°C. The agar disc was weighed carefully for confirming exact amount of the extract and isolated pure compounds being incorporated (compared to preweighed blank discs).

#### 2.6.2. Bacterial Culture

*Escherichia coli* and *Pseudomonas aeruginosa* which were isolated from stool specimens in the clinic were identified according to routine cultural properties and biochemical tests. Four strains of each were included in the study. A few colonies from the overnight culture of Eosin Methylene Blue (EMB) agar were transferred into approximately 4-5 mL Trypticase soy broth (TSB) medium. The broth was incubated at 37°C for 3-4 hr, and the turbidity of suspension was adjusted to that of 0.5 McFarland barium sulfate standards. The standard suspension was used for both qualitative and quantitative antibacterial assays.

#### 2.6.3. Bacterial Susceptibility Testing

Standardized inoculums (20 *µ*g/mL) were introduced on to the surface of sterile agar plates, and a sterile glass spreader was used for even distribution of the inoculums. Sterile agar-disc diffusion previously soaked in a known concentration of extract or pure compound (20 *µ*g/mL per disc) was carefully placed at the center of the labeled seeded plate. The same procedure was used for all the MRSA strains used. The plates were incubated aerobically at 37°C and examined for zones of inhibition after 24 hr. The inhibition zones were measured with a ruler and compared with the control disc (disc containing only physiological saline). Strains of human pathogen microorganisms used in this study were as follows: two Gram-negative bacteria, *Pseudomonas aeruginosa*, *Escherichia coli*, and two Gram-positive bacteria *Staphylococcus aureus* and *Bacillus subtilis*.

The bacterial stock cultures were incubated for 24 hr at 37°C on nutrient agar medium (Oromia Public Health Research Laboratory, Adama). The bacterial strains were grown in the Mueller–Hinton agar (MHA) plates at 37°C (the bacteria were grown in the nutrient broth at 37°C and maintained on nutrient agar slants at 4°C). The agar was melted (50°C), and the microorganism cultures were then added aseptically to the agar medium at 45°C in plates and poured into sterile petri dishes to give a solid plate. All these experiments were performed in duplicate. The plates were incubated for 24–48 hr at 37°C for bacteria.

The inhibition zones produced by the plant extracts were compared with the inhibition zones produced by commercial standard antibiotics. One dilution (20 *µ*g/mL) of *Clausena anisata* extract, pure compound, and standard drugs was prepared in DMSO using nutrient agar tubes. Mueller–Hinton sterile agar plates were seeded with indicator bacterial strains (108 cfu) and allowed to stay at 37°C for 3 hr. Control experiments were carried out under similar conditions by using gentamicin for antibacterial activity as a standard drug. The zones of growth inhibition around the disks were measured after 24 hr of incubation at 37°C for bacteria. The sensitivities of the microorganism species to the plant extract and isolated pure compounds were determined by measuring the sizes of inhibitory zones (including the diameter of disk) on the agar surface around the disks, and values <6 mm were considered as not active against microorganisms [1, 9].

## 3. Results and Discussion

### 3.1. Phytochemical Screening

Phytochemical screening test of dichloromethane/methanol (1 : 1) and methanol roots extracts revealed the presence of flavonoids, phytosterols, coumarins, phenols, alkaloids, tannins, terpenoids, and free reducing sugars and the absence of saponins (Table 1).

### 3.2. Structure Elucidation of Compounds

Compound **1** was obtained as a brown powder (melting point: 227–228°C) with an *R*_f_ value of 0.52 in *n*-hexane/EtoAc (8 : 2) as eluent. The IR (KBr disk) spectrum showed broad vibration at 3290 cm^−1^ attributed to hydroxyl (OH) and sharp absorption at 1608 cm^−1^ attributed to a carbonyl moiety. The ^1^H NMR spectrum (CDCl_3_, 400 MHz, Table 2) showed signals for a singlet proton at *δ* 11.68 (1H, *s*, OH) indicative of the hydroxyl (OH) group. The downfield chemical shift of the hydroxyl group suggests the presence of intermolecular hydrogen bonding (*Peri* effect), the presence of singlet peak at *δ* 9.94 (1H, *s*, CHO) aldehyde proton. The presence of four aromatic protons observed at *δ* 7.28 (1H, *dd*, H-6), 7.43 (1H, *dd*, H-7), 7.95 (1H, *dd*, H-5, *J* = 7.75 Hz), and 7.99 (1H, *dd*, H-8) indicates the presence of disubstituted aromatic ring. The presence of two aliphatic methyl protons at *δ* 1.91 (3H, *s*, H-4′) and *δ* 1.80 (3H, *s*, H- 5′), olefinic proton at *δ* 5.35 (1H, *t*, *J* = 5.96 Hz), and benzylic methylene protons at *δ* 3.66 (*d*, H-1′ *J* = 6.90 Hz) suggests the presence of a prenyl group in the compound.

Moreover, the presence of a downfield singlet aromatic proton at *δ* 8.07 suggests that this proton is experiencing anisotropic effect of the aldehyde carbonyl. The above chemical shift positions for the aromatic singlet proton (H-4) and downfield chemical shift of hydroxyl moiety allow for unequivocal assignment of aldehyde moiety at C-3 between two carbons bearing proton H-4 (C-4) and that of C-2 bearing hydroxyl group. The ^13^ C NMR spectrum (CDCl_3_, 100 MHz, Table 2) in combination with DEPT-135 showed the presence of 18 carbons. Among these, six signals are due to methine carbons, eight quaternary, one benzylic methylene, two methyls, and one carbonyl. The peak at *δ* 195.4 is due to the carbonyl group of aldehyde moiety. Oxygenated sp^2^ quaternary carbon was observed at *δ* 157.9 (C-2). The remaining carbons of the aromatic methine carbons were observed at *δ* 125.9 (C-4), 119.8 (C-5), 123.7 (C-6), 120.9 (C-7), and 110.9 (C-8). Furthermore, the spectrum displayed signals due to quaternary carbons at *δ* 109.1 (C-1), 115.5 (C-3), 117.4 (C-4a), 125.3 (C-4b), 134.2 (C-3′), 140.2 (C-8a), and 145.1 (C-9a). The prenyl moiety appeared at *δ* 22.8 (C-1′), 121.3 (C-2′), 134.2 (C-3′), *δ* 25.9 (C-4′), and 18.2 (C-5′). Thus, based on the above spectral data and comparison with literature, the structure of compound **1** was found to be a derivative of carbazole alkaloid (**1**) known by the trivial name heptaphyline [10], where the later has a hydroxyl group at C-7 position.

Compound **2** was obtained as a brown crystalline powder with an *R*_f_ value of 0.56 in the *n*-hexane/EtoAc (7 : 3) solvent system. The IR (KBr disk) spectrum showed broad vibration at 3419 cm^−1^ due to the presence of the hydroxyl moiety. The strong sharp vibrations at 1617 cm^−1^ and 1100 cm^−1^ suggest the presence of olefinic C=C and carbon-oxygen (C-O), respectively. Moreover, intense vibrations at 2849 cm^−1^and 2930 cm^−1^indicate C-H vibrations of methylene (sp^2^) and methyls (sp^3^), respectively.

The ^1^H NMR spectrum (CDCl_3_, 400 MHz, Table 3) revealed peaks at *δ* 7.54 (1H, *s*, H-4), *δ* 7.193 (1H, s, H-6), and *δ* 6.955 (1H, s, H-9) attributed to aromatic protons. The presence of prenyl moiety was confirmed on the basis of methylene protons (H-2″) adjacent to olefinic carbon; olefinic proton (H-3″) and two methyl groups were observed at *δ* 3.38, 5.34 (1H, *t*, H-3″) and *δ* 1.78 (3H, *s*, H-5″) and 1.81(3H, *s*, H-6″), respectively. The peak at *δ* 6.18 (1H, *dd*, *J* = 10.8, 17.6 Hz, H-2′) attributed to olefinic proton adjacent to terminal olefinic methylene protons at *δ* 5.09 (1H, H-3″) and *δ* 5.07 (1H, H-3″). Symmetrical methyl protons were observed at *δ* 1.48 (3H, s, H-4′, 5′). The presence of two singlet aromatic protons at *δ* 7.19 (1H, s, H-6) and *δ* 6.95 (1H, s, H-9) coupled with a downfield proton at *δ* 7.54 (1H, *s*) is in good agreement with a chromene moiety, where the latter (H-4) is located at *β*-position of the lactone moiety, whereas H-6 and H-9 are located at 1,4-positions of the aromatic ring of chromene skeleton.

The ^13^C NMR (CDCl_3_ 100 MHz, Table 3) spectrum in combination with DEPT-135 showed a resonance for 18 carbon atoms. Among these, five signals are due to methine carbons, eight quaternary, three methyl, and two methylene carbons. The most downfield signals appearing at *δ*160.7 attributed to the ester carbonyl, whereas the quaternary carbons appearing at *δ*157.3(C-8) and *δ*153.3 (C-10) were assigned to sp^2^ oxygenated quaternary carbons. Methine aromatic carbons were observed at *δ* 138.3 (C-4), 102.5 (C-9), 128.2 (C-6), *δ* 121.2 (C-3″), and 145.6 (C- 2′). The methyl signals due to C-5″ and C-6″ were observed at *δ* 25.8 and 17.9, respectively. Symmetrical carbon signals were also observed for C-4′ and C- 5′at *δ* 26.2, whereas the methylene signals were observed at 28.6 (C-2″) and 112.8 (C-3′). Furthermore, the spectrum displayed signals due to quaternary carbons at *δ* 131.2, 112.0, 124.9, 135.0, and 40.3 assigned to C-3, C-5, C-7, C-4″, and C-1′, respectively. Thus, based on the above spectral data and comparison with literature, the structure of compound **2** was proposed to be a chromene skeleton as shown below.

Compound **3** was isolated as orange powder with an *R*_f_ value of 0.59 in *n*-hexane/EtoAc (6 : 4) as solvent system. The IR (KBr disk) spectrum showed broad absorption band at 1723 cm^−1^, 1297 cm^−1^, and 2917 cm^−1^ attributed to C=C stretching of aromatic ring, C-O stretching, and C-H stretching of methyl group, respectively. The ^1^H NMR (CDCl_3_, 400 MHz, Table 4) spectrum showed two proton doublets at *δ* 6.36 (1H, *d*, *J* = 9.6 Hz)and 7.77 (1H, *d*, *J* = 9.6 Hz) attributed to olefinic protons, of which one of them is downfield due to *β*-position of the lactone moiety. Two olefinic protons were observed at *δ* 7.69 (1H, *d*, H-2″, *J* = 2.4 Hz) and 6.82 (1H, *d*, H-3″, *J* = 2.4 Hz) coupling to each other, suggesting the presence of a furan ring attached to the aromatic ring. The presence of singlet aromatic proton was observed at *δ* 7.36 (1H, *s*, H-6). The presence of prenyl group was confirmed based on peaks of two methyl signals at *δ* 1.72 (3H, *s*, H-4′) and *δ* 1.74 (3H, *s*, H-5′), olefinic proton at *δ* 5.65 (1H, *t*, H-2′), and oxygenated methylene at *δ* 5.0 (2H, *t*). The latter suggests that the prenyl group is attached to oxygen.

Moreover, the above ^1^H NMR pattern suggests the compound has chromene skeleton with a furan ring fused to it. The ^13^ C NMR (CDCl_3_, 100 MHz, Table 4) spectrum showed a total of sixteen carbon atoms. The downfield chemical shift signal that appeared at *δ* 160.5 coupled with signals at *δ* 114.6 and 143.8 suggest *α*,*β*-conjugated lactone moiety. The other five quaternary carbons at *δ* 116.5, 125.9, 131.6, 144.5, and 148.6 were assigned to C-5, C-7, C-9, C-10, and C-8, respectively. Of these, three of the carbons are sp^2^-oxygenated quaternary carbons, that is, C-8, C-9, and C-10 of aromatic ring.

The methine carbons of the furan moiety were observed at *δ* 146.6 (C-2″) and 106.7 (C-3″), of which the downfield chemical shift value of the former is in agreement with its attachment to the oxygen atom. The aromatic methane at C-6 appeared at *δ* 113.2. Oxygenated methylene of the prenyl moiety appeared at *δ* 70.2 (also confirmed by DEPT-135 pointing downwards), whereas the remaining carbons of prenyl moiety group appeared at *δ* 119.8 (C-2′), 139.7 (C-3′), 25.8, and 18.2 (C-4′ and C-5′), respectively. Thus, based on the above spectral features, compound **3** was found to be in good agreement with a chromene skeleton known by trivial name imperatorin (**3**) [12].

Compound **4** was obtained as a yellowish amorphous powder (melting point 175–176°C) with an *R*_f_ value of 0.53 in *n*-hexane/ethyl acetate (4 : 6) solvent system. The IR (KBr disk) spectrum showed broad vibration at 3385 cm^−1^, sharp absorptions at 1600 cm^−1^ and 1255 cm^−1^ attributed to hydroxyl moiety (OH), aromatic benzene ring, and C-O stretching, respectively. The strong absorption band at 2925 cm^−1^ showed the presence of the C-H stretching of sp^3^ aliphatic moiety. The absorption band at 1730 cm^−1^ showed the presence of the C=O stretching of carboxyl moiety.

The ^1^H NMR spectrum (CDCl_3_, 400 MHz, Table 5) revealed the presence of aromatic protons at *δ* 7.178 (1H, *s*, H-5) and 6.69 (1H, *s*, H-8), suggesting two para oriented aromatic protons, whereas downfield chemical shift of proton at *δ* 7.48 (1H, *s*, H-4) suggest the *β*-position of the *α*,*β*-conjugated system. The peaks at *δ* 3.19 (1H, *s*, H-3′) and *δ* 3.17 (H, *s*, H-3′) suggest the presence of diastereotopic methylene protons adjacent to asymmetric carbon (C-2′). This coupled with the presence of oxygenated methine signal at *δ* 4.73 (1H, *t*) suggests the presence of furan ring. Methyl signals were observed at *δ* 1.47 (6H, *s*, H-4″, 5″), *δ*1.37 (3H, *s*, H-5′), and *δ* 1.27 (3H, *s*, 6′). The presence of terminal olefinic protons at *δ* 5.09 (2H, *dd*, H-3″) coupled with olefinic proton at *δ* 6.17 suggests the presence of rearranged prenyl moiety in the compound. The above ^1^H NMR pattern suggests that the compound has a coumarin skeleton where a reduced furan ring moiety is fused to the aromatic ring and *α*,*β*-conjugated lactone ring bearing the rearranged prenyl group.

The ^13^C NMR spectrum (CDCl_3_, 100 MHz, Table 5) revealed a total of eighteen carbon signals, of which the downfield peak at *δ* 162.3 is attributed to the ester carbonyl group, whereas the sp^2^-oxygenated quaternary aromatic carbons appeared at *δ* 160.3 (C-7) and *δ* 154.7 (C-9). The signal at *δ* 71.7 (C-4′) was assigned to the oxygenated sp^3^ quaternary carbon. The signal for remaining quaternary carbons were observed at *δ* 130.8 (C-3), 124.3 (C-6), 113.1(C-10), and 40.3 (C- 1″). Methine carbons appeared at *δ*138.1, 123.4, 97.2, 90.0, and 145.8 were assigned to C- 4, C-5, C-8, C-2′, and C-2″, respectively. Furthermore, the spectrum displayed signals due to methylene carbons at *δ* 29.7 and *δ* 112.1 were assigned to C-3′ and C-3″, also confirmed by DEPT-135 pointing downwards. Symmetrical carbon signals were observed for C-4″ and 5″at *δ* 26.1 and remaining methyl signals were also observed at *δ*26.0 for C-5′ and 24.3 (C-6′). Thus, based on the above spectral features, compound **4** was found to be in good agreement with a chromene skeleton known by the trivial name chalepin (**4**) [13].

### 3.3. Antibacterial Activity

The antibacterial activity of the extract and isolated compounds of *C. anisata* were examined at a concentration of 20 *μ*g/mL against four pathogenic bacterial strains: two Gram-positive bacteria, *S. aureus* and *B. substilis*, and two Gram-negative bacteria, *E. coli* and *P. aeruginosa* (Table 6). The results revealed that the derivative of heptaphyline (**1**) and imperatorin **(3**) exhibited comparable antibacterial activity against *S. aureus* and *B. substilis*, and a 14-mm zone of inhibition for both strains, compared with that of ciprofloxacin (15 mm). Chalepin (**4**) also exhibited promising antibacterial activity against *S. aureus* and *P. aeruginosa* with 14 and 12 mm zone of inhibition, respectively, compared with that of ciprofloxacin (15 mm), whereas chalepin (**4**) revealed more antibacterial activity (16 mm zone of inhibition) against *B. substilis* compared with that of ciprofloxacin (15 mm). Generally, crude extracts and pure isolated compounds were ineffective against *E. coli* pathogen at this concentration.

## 4. Conclusion

This study is one of the few attempts to isolate phytochemical constituents from the roots of *C. anisata* of Ethiopian biodiversity. Phychemical screening of the dichloromethane/methanol (1 : 1) and methanol roots extracts revealed the presence of flavonoids, phytosterols, coumarins, phenols, alkaloids, tannins, terpenoids, and free reducing sugars and the absence of saponins. Silica gel column chromatographic separation of the dichloromethane/methanol (1 : 1) and methanol roots extracts gave three coumarins (**2-4**) including the known coumarins imperatorin (**3**) and chalepin (4) along with a carbazole alkaloid (**1**), derivative of known carbazole alkaloid heptazoline. Compounds **2** and **4** isolated for the first time from the roots of *C. anisata*. The structures of the compounds were characterized on the basis of spectral data (^1^H NMR, ^13^C NMR, DEPT-135, and IR) as well as in comparison with the literature report. In agreement with the previous study, the wide traditional use of the plant may be attributed to its rich alkaloids and coumarin constituents. The antibacterial test results revealed that the isolated compounds showed promising antibacterial activity against *S. aureus*, *P. aeruginosa*, and *B. substilis*. Derivative of heptaphyline (**1**) and imperatorin (**3**) exhibited promising antibacterial activity against *S. aureus* and *B. substilis* (14 and 13 mm zone of inhibition, respectively) compared with ciprofloxacin (15 mm at a concentration of 20 *µ*g/mL). Chalepin (**4**) revealed more antibacterial activity against *B. substilis* (16 mm zone of inhibition) compared with ciprofloxacin (15 mm zone of inhibition). The finding of these pharmacologically important secondary metabolites from roots extracts brings the attention of researchers to do more research work on the medicinal importance of the plant.

## Figures and Tables

**Table 1:** Phytochemical screening test results.

Secondary metabolite	CH_2_Cl_2_/CH_3_OH (1 : 1) extract	MeOH extract
Coumarins	+	+
Saponins	−	−
Terpenoids	+	+
Phytosterols	+	+
Flavonoids	+	+
Alkaloids	+	+
Phenols	+	+
Tannins	+	+
Free reducing sugars	+	+

+ indicates presence; − indicates absence.

**Table 2:** ^1^H-NMR (CDCl_3_, 400 MHz), ^13^C-NMR, and DEPT-135 (100 MHz) spectral data of compound **1**.

Position	^1^H NMR	^13^C NMR	DEPT-135	[10]	
				^1^H NMR	^13^C NMR
CHO	9.94, *s*	195.4	195.4	9.90, *s*	196
2(−OH)	11.68, *s*	157.9	—	—	156.3
NH	8.19, *s*	—	—	—	—
8a	—	140.2	—	8.25, *s*	142.3
9a	—	145.1	—	—	144.8
3	—	115.5	—	—	114.7
4	8.05, *s*	125.9	125.9	—	124.3
5	7.95 (1H, *d*, *J* = 7.75, 2.1 Hz)	119.8	119.8	8.00, *d*	120.5
4b	—	125.3	—	—	116.3
6	7.41, *s*	123.7	123.7	—	108.5
7	7.43 (1H, *dd*, *J* = 7.75, 2.1 Hz)	120.9	120.9	—	158.5
8	7.99 (1H, *dd*, *J* = 8, 2.1 Hz)	110.9	110.9	—	95.6
4a	—	117.4	—	—	117.0
1	—	109.1	—	—	108.9
1′	3.66, (1H, *d*, *J* = 6.90 Hz)	22.9	22.9	3.65, *d*, *J* = 7 Hz	22.6
2′	5.35, 1H (*t*)	121.3	121.3	5.35, *t*, *J* = 6 Hz	121.6
3′	—	134.2	—	—	131.7
4′	1.91, *s*	25.7	25.8	1.66, *s*	25.7
5′	1.80, *s*	18.2	18.2	1.82, *s*	17.9
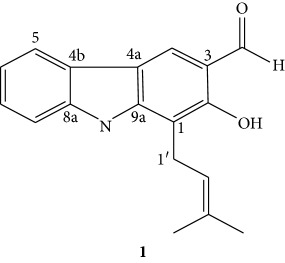					

**Table 3:** The ^1^H NMR (CDCl_3_, 400 MHz), ^13^C NMR (CDCl_3_, 100 MHz), and DEPT-135 spectral data of compound **2**.

Position	^1^H NMR	^13^C NMR	DEPT-135	[11]	
				^1^H NMR	^13^C NMR
2	—	160.7		—	161.1
3	—	131.3		—	131.9
4	7.54, 1H, *s*	138.3	138.29	7.54, 1H, *s*	138.5
5	—	112.0		—	112.1
6	7.19, 1H, *s*	128.2	128.16	7.17, 1H, *s*	128.2
7	—	124.9		—	125.4
8	—	157.3		—	157.5
9	6.95, 1H, *s*	102.5		7.04, 1H, *s*	102.5
10	—	153.3		—	153.2
1′	—	40.3		—	40.31
2′	6,16, (1H, *dd*, *J* = 10.8, 17.6)	145.6	145.64	6.16, 1H, *dd*, *J* = 10.2, 18 Hz	145.6
3′	5.09, 5.07 (2H, *dd*, *J* = 10.2, 18 Hz)	112.8	112.77	5.1 (2H, *dd*, *J* = 10.2, 18 Hz)	112.7
4′,5′	1.48, 6H, *s*	26.2		1.48 (6H, *s*)	26.1
2″	3.38 (2H, *d*, *J* = 7.2 Hz)	28.6	28.62	3.38 (2H, *d*, *J* = 7.2 Hz)	28.4
3″	5.33 (1H, *t*)	121.2		5.33, 1H, *m*	121.3
4″	—	135.0		—	134.6
5″	1.80 (3H, *s*)	25.8	25.83	1.80, 3H, *s*	25.8
6″	1.78 (3H, *s*)	17.9	17.89	1.75, 3H, *s*	17.9
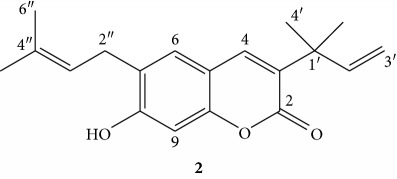					

**Table 4:** ^1^H NMR (CDCl_3_, 400 MHz), ^13^C NMR, and DEPT-135 (100 MHz) spectral data of compound **3**.

Position	^1^H NMR	^13^C NMR	DEPT-135	[12]	
				^1^H NMR	^13^C NMR
2	—	160.5	—		160.6
3	6.36, 1H, *d* (*J* = 9.6 Hz)	114.6	114.6	6.36, *d*	113.0
4	7.77, 1H, d (*J* = 9.4 Hz)	143.8	143.8	7.75, *d*	144.0
5	—	116.5			116.4
6	7.36, 1H, *s*	113.2	113.2	7.35, *s*	114.8
7	—	125.8	—	—	126.0
8	—	148.6	—	—	148.6
9	—	131.6	—	—	132.0
10	—	144.4	—	—	143.8
2″	7.69, 1H, *d (J* = 2.4)	146.6	146.7	7.68, *d*	146.6
3″	6.82, 1H, *d* (*J* = 2.05)	106.7	106.7	6.82, *d*	106.7
1′	5.00, 2H, *d* (*J* = 7.10)	70.2	70.2	4.95, *d*	69.9
2′	5.61, 1H, *t* (7.35)	119.8	119.8	5.61, *t*	119.6
3′	—	139.7	—	—	139.7
4′	1.72, 3H, *s*	25.8	25.8	1.68, *s*	25.9
5′	1.74, 3H, *s*	18.1	18.1	1.73, *s*	18.2
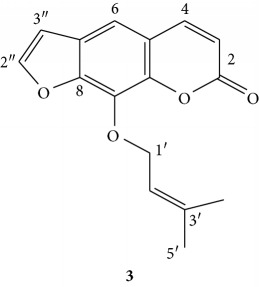					

**Table 5:** ^1^H NMR (CDCl_3_, 400 MHz), ^13^C NMR, and DEPT-135 (100 MHz) spectral data of compound **4**.

Position	^1^H NMR	^13^C NMR	DEPT-135	[13]	
				^1^H NMR	^13^C NMR
2	—	162.3	—	—	162.3
3	—	130.8	—	—	130.9
4	7.48 (1H, *s*, H-4)	138.1	138.1	7.48, 1H, *s*	138.1
5	7.17 (1H, *s*, H-5)	123.3	123.25	7.20, 1H, *s*	123.3
6	—	124.6	—	—	124.6
7	—	160.3	—	—	160.2
8	6.68 (1H, *s*, H-8)	97.1	97.1	6.71, 1H, *s*	97.1
9	—	154.6	—	—	154.6
10	—	113.1	—	—	113.1
2′	4.73 (1H, *dd*, *J* = 10, 6 Hz)	90.9	90.9	4.72 (1H, *t*, *J* = 12, 6 Hz)	90.9
3′	3.19 (2H, *dd*, *J* = 18, 8 Hz)	29.7	29.7	3.21 (2H, *dd*)	29.6
4′	—	71.7	—	—	71.7
5′	1.37 (3H, *s*)	26.0	26.02	1.37 (3H, *s*)	26.0
6′	1.27 (3H, *s*)	24.21	24.21	1.27 (3H, *s*)	24.2
1″	—	40.3	—	—	40.3
2″	6.17 (1H, *dd*, *J* = 10.8, 6.4 Hz)	145.6	145.6	6.17, 1H, *dd*, *J* = 18, 12 Hz)	145.6
3″	5.10 (2H, *m*, H-3″)	112.1	112.1	5.09 (2H, *dd*)	112.1
4″, 5″	1.47 (6H, *s*, H-4″, 5″)	26.2	26.2	1.47 (6H, *s*)	26.11
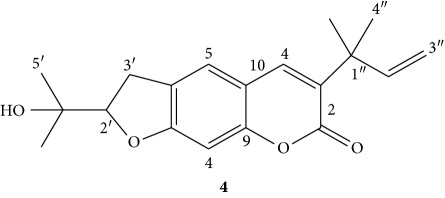					

**Table 6:** Zone of bacterial growth inhibition (mm) for crude extract and isolated compounds.

Sample	*E. coli*	*S. aureus*	*B. substilis*	*P. aeruginosa*
CH_2_Cl_2_/MeOH extract	9 ± 0.1	11 ± 0.1	10 ± 0.1	8 ± 0.1
MeOH extract	*n*	12 ± 0.2	13 ± 0.3	9 ± 0.2
**1**	*n*	14 ± 0.1	12 ± 0.1	12 ± 0.1
**3**	*n*	13 ± 0.1	14 ± 0.1	14 ± 0.1
**4**	*n*	14 ± 0.2	16 ± 0.3	12 ± 0.2
Ciprofloxacin	14 ± 0.1	15 ± 0.3	15 ± 0.3	15 ± 0.3

*n* ≤ 6 is null, and *n* > 6 is sensitive.

## Data Availability

The data used to support the findings of this study are available from the corresponding author upon request.

## Supplementary Materials

Supplementary Materials IR and NMR spectral data of compounds **1**–**4** are freely available along with the manuscript as supplementary material.
