# Supplementary material for: Antibacterial Activity of Coumarins and Carbazole Alkaloid from Roots of Clausena anisata
Source: Adv Pharmacol Sci. 2019 Feb 3;2019:5419854. doi: 10.1155/2019/5419854 (PMC6378073; doi:10.1155/2019/5419854)

Appendix 1: IR spectrum of compound **1**

Appendix 2: ^1^H-NMR spectrum of compound **1**


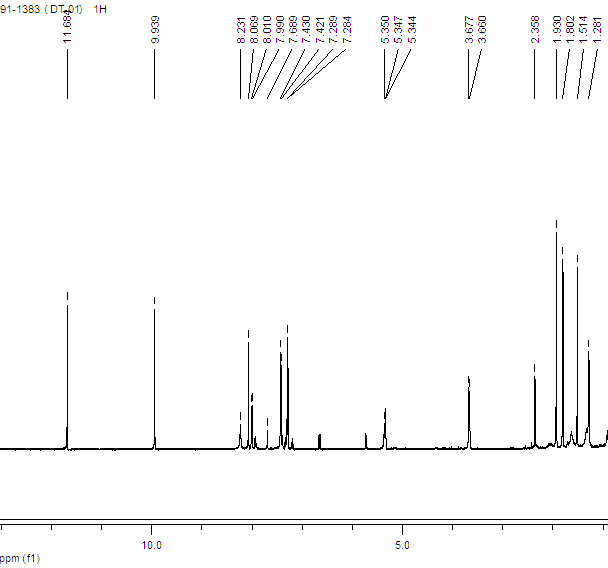


Appendix 3: ^13^C-NMR spectrum of compound**1**


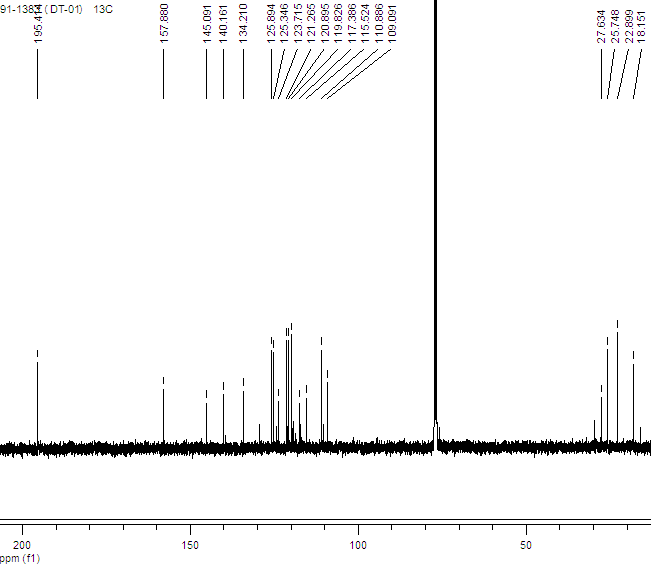


Appendix 4: DEPT-135 of compound **1**


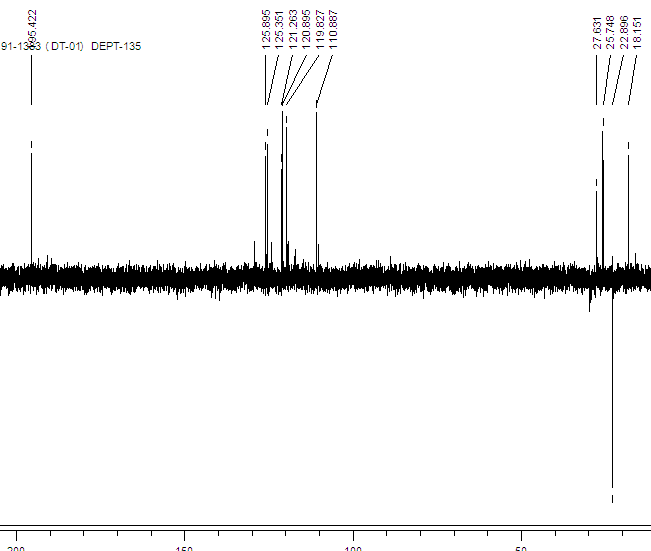


Appendix 5: IR spectrum of compound-**2**

Appendix 6: ^1^H-NMR spectrum of compound**2**


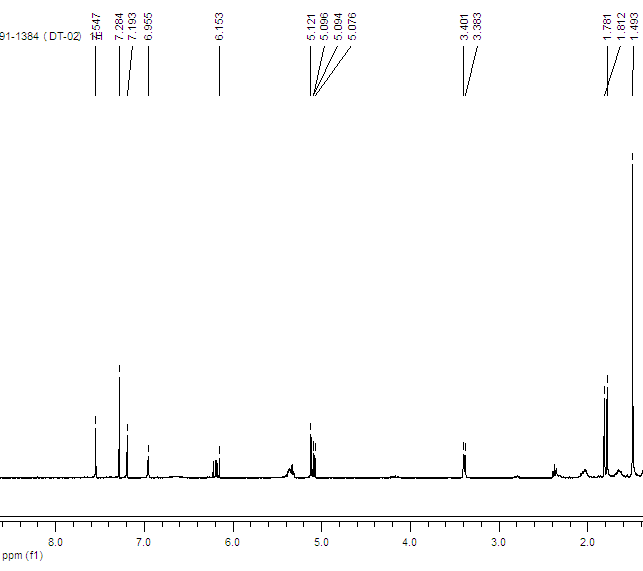


Appendix 7: ^13^C- NMR spectrum of compound **2**


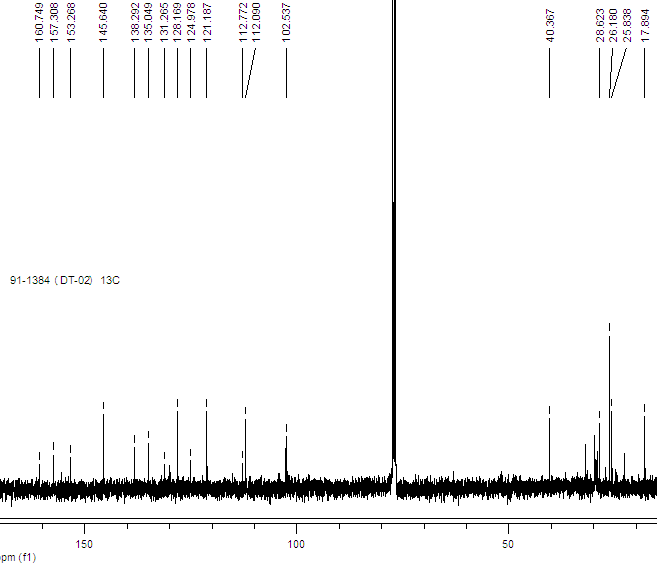


Appendix 9: IR spectrum of compound-**3**

Appendix 10: ^1^H-NMR spectrum of compound **3**


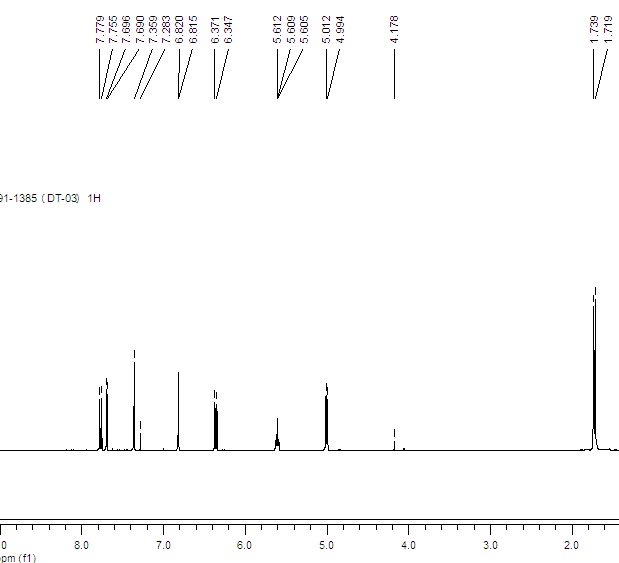


Appendix 11: ^13^C- NMR spectrum of compound **3**


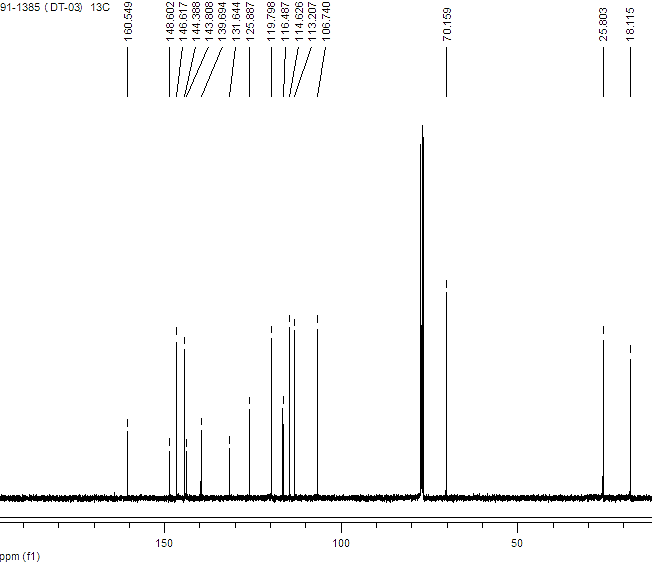


Appendix 12: DEPT-135 of compound **3**


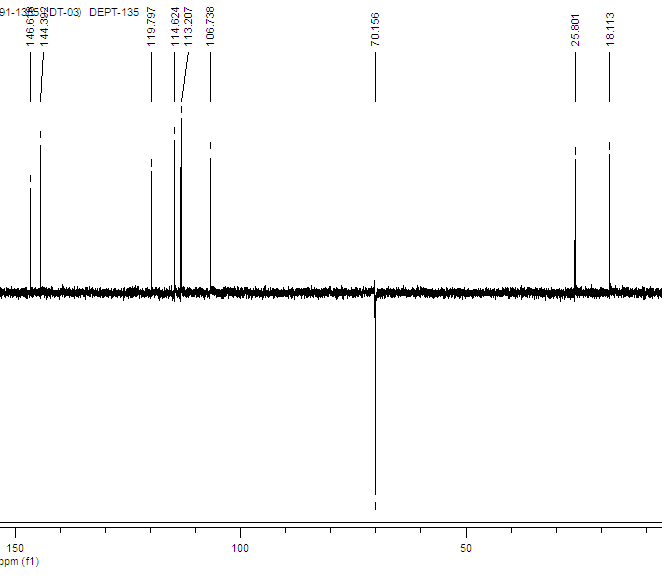


Appendix 13: IR spectrum of compound-**4**

Appendix 14: ^1^H-NMR spectrum of Compound **4**


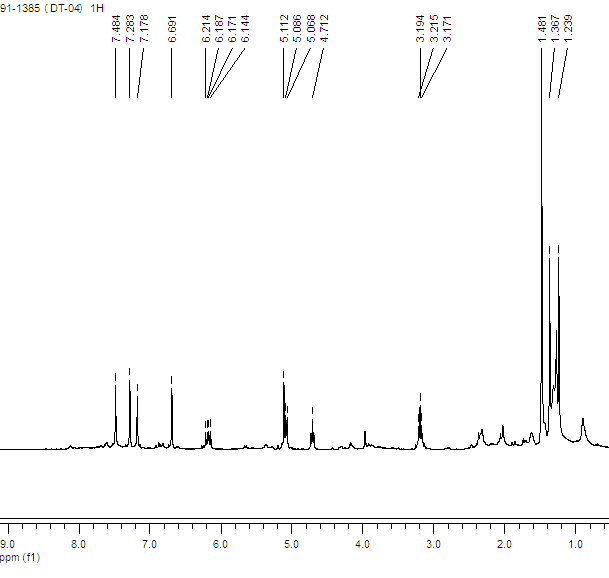


Appendix 15: ^13^C- NMR spectrum of compound **4**


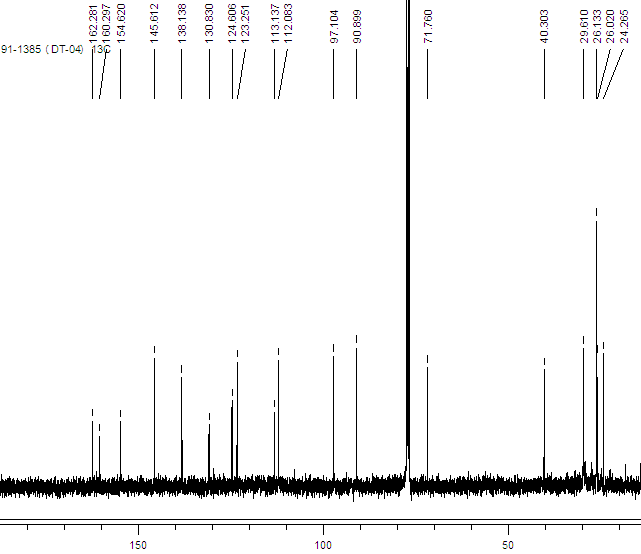


Appendix 16: DEPT-135 of Compound **-4**


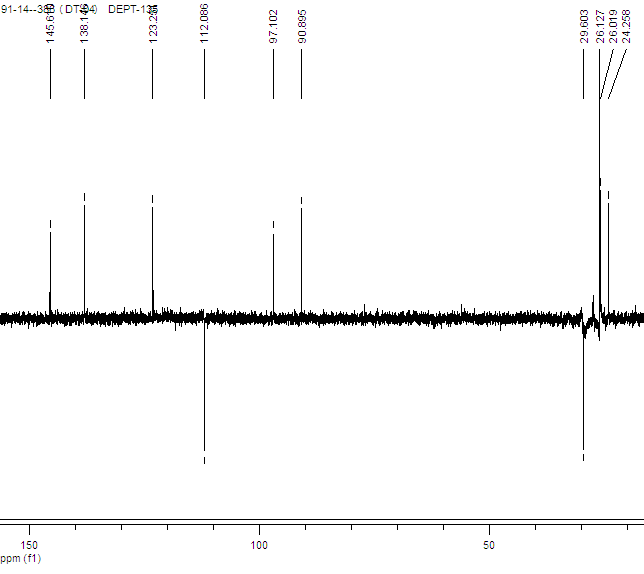

Supplement: Supplementary Materials — IR and NMR spectral data of compounds 1–4 are freely available along with the manuscript as supplementary material. [file 5419854.f1.docx]
